# Targeting Psychotic and Cognitive Dimensions in Clinical High Risk for Psychosis (CHR-P): A Narrative Review

**DOI:** 10.3390/jcm14155432

**Published:** 2025-08-01

**Authors:** Michele Ribolsi, Federico Fiori Nastro, Martina Pelle, Eleonora Esposto, Tommaso B. Jannini, Giorgio Di Lorenzo

**Affiliations:** 1 Department of Life Science, Health, and Health Professions, Link Campus University, 00165 Rome, Italy; m.ribolsi@unilink.it; 2 Department of Systems Medicine, Tor Vergata University of Rome, 00133 Rome, Italy; m.pelle@policlinicocampus.it (M.P.); eleonora.esposto@gmail.com (E.E.); t.jannini@med.uniroma2.it (T.B.J.); di.lorenzo@med.uniroma2.it (G.D.L.); 3 IRCCS Fondazione Santa Lucia, 00179 Rome, Italy; 4 Unit of Neurology, Neurophysiology, Neurobiology and Psychiatry, Department of Medicine, Campus Bio-Medico University, 00128 Rome, Italy

**Keywords:** antipsychotics, UHR, CHR-P, clinical high risk of psychosis, ultra-high risk of psychosis, positive symptoms, negative symptoms, cognitive abilities

## Abstract

Schizophrenia (SCZ) is a debilitating disorder with substantial societal and economic impacts. The clinical high risk of psychosis (CHR-P) state generally precedes the onset of SCZ, offering a window for early intervention. However, treatment guidelines for CHR-P individuals remain contentious, particularly regarding antipsychotic (AP) medications. Although several studies have examined the effects of APs on reducing the risk of conversion to psychosis, the novelty of this narrative review lies in its focus on differentiating APs’ effects on positive and negative symptoms, as well as cognitive functioning, in CHR-P individuals. Evidence suggests that APs may be cautiously recommended for attenuated positive symptoms to stabilize individuals for psychological interventions, but their use in treating negative symptoms is generally discouraged due to limited efficacy and potential side effects. Similarly, the effects of APs on cognitive abilities remain underexplored, with results indicating a lack of significant neurocognitive outcomes. In conclusion, APs’ use in CHR-P patients requires careful consideration due to limited evidence and potential adverse effects. Future research should focus on individual symptom domains and treatment modalities to optimize outcomes in this critical population. Until then, a cautious approach emphasizing non-pharmacological interventions is advisable.

## 1. Introduction

Schizophrenia (SCZ) is a life-long, debilitating psychotic disorder leading to reduced quality of life and life expectancy and represents a major economic burden for the individual, their family, and society [1,2,3]. Before the onset of a frank psychosis, most individuals experience a prodromal phase of sub-clinical and subjective symptoms [4,5]. The worldwide research conducted over the past 30 years on early detection has resulted in operationalizing the psychosis prodrome within the construct of the clinical high risk for psychosis (CHR-P). Two complementary approaches are currently the primary methods used to characterize the CHR-P state [6]: the ultra-high risk (UHR) criteria [7], and the basic symptoms criteria (BS) [8]. Most high-risk centers now include individuals with UHR and/or BS in their studies to identify subjects at an enhanced risk of developing a first-episode psychotic disorder (FEP). The UHR subgroups were defined as “attenuated psychotic symptoms” (APSs) syndrome, “brief, limited, intermittent psychotic symptoms” (BLIPSs), and “genetic risk and functioning deterioration” (GRFD) syndrome [9,10,11]. The basic symptoms model focuses on subtle, self-perceived disturbances in affective, perceptual, and cognitive processes that may precede and contribute to the development of full-blown psychotic symptoms [12].

The emerging paradigm of early detection in psychosis has led to the inclusion of Attenuated Psychosis Syndrome in the Diagnostic and Statistical Manual of Mental Disorders, Fifth Edition (DSM-5) [13], where it is listed in the section on conditions warranting further investigation.

CHR-P individuals may present with a heterogeneous symptom profile, including subthreshold positive symptoms (e.g., perceptual disturbances, suspiciousness), negative symptoms, and cognitive impairments. These may be accompanied by nonspecific affective disturbances—such as low mood, anxiety, and irritability—as well as a functional decline in academic, occupational, or social domains [10].

The early identification of a high-risk mental state offers a critical opportunity for timely intervention, potentially preventing the development of a full-blown chronic disorder, which is often associated with complex treatment needs and poor clinical outcomes. Recent evidence highlights that approximately 15% of CHR-P individuals transition to a psychotic disorder within one year, with rates rising to 35% over a 10-year period [14].

However, due to the non-specific nature of subclinical symptoms, accurate identification of CHR-P and treatment remain challenging. Various treatments have been proposed, including psychosocial interventions, psychotherapies, APs, antidepressants, and social support strategies [15,16]. The use and timing of pharmacological treatment in CHR-P individuals remain controversial [17,18,19]. Ethical considerations regarding high false positive rates and adverse reactions related to pharmacotherapy are of concern [20,21].

However, the use of APs in CHR-P individuals is relatively frequent in clinical settings, with baseline prevalence rates reported between 23% and 77% across studies [22]. A recent systematic review [23] reported that only 36.8% of studies excluded baseline AP exposure, while over 60% allowed it, leading to AP use in nearly 25% of CHR-P participants at study entry. This finding is notable, as it contrasts with current treatment guidelines that recommend a cautious approach and advise using APs only after non-pharmacological interventions have been exhausted [16]. To date, Australian, Canadian, and European guidelines acknowledge the use of APs under certain circumstances, while English guidelines do not recommend their use at all [6,24,25].

Since adequate treatment is fundamental for limiting the conversion rate, the topic has been widely investigated with different results. Recent evidence showed that CHR-P with AP prescription at baseline has higher psychosis transition rates compared with those without APs, although the underlying cause remains unclear [19,26,27,28,29].

Several studies have analyzed the effects of APs in terms of reducing the risk of conversion to frank psychosis, but few have focused on differentiating their effects on positive, negative, and cognitive symptoms.

This narrative review aimed to summarize current evidence on the effects of AP treatment in CHR-P individuals across three key clinical dimensions: positive symptoms, negative symptoms, and cognitive impairments.

Since research on the CHR-P paradigm began in the late 1990s, we reviewed the literature published over the past 25 years (2000–2025) across major electronic databases, including PsycInfo, PubMed, Web of Science, and Scopus. The final search was conducted immediately prior to the submission of this manuscript (June 2025).

## 2. Antipsychotics’ Effects on Positive Symptoms in CHR-P Individuals

A large proportion of CHR-P individuals present with subthreshold or threshold positive symptoms and may require some form of intervention. As previously noted, APs are not considered a first-line treatment [30] due to their potential side effects and associated stigma. Their use is generally reserved for cases of rapid clinical deterioration, increased risk of self-injury, aggression, or hostility. Additionally, according to the European Psychiatric Association (EPA) guidance [16], APs may be considered to prevent the development or persistence of social, educational, or vocational impairments. When psychological interventions have been ineffective, the use of second-generation antipsychotics (SGAs) at low doses has to be considered [16].

Numerous studies, particularly in the early 2000s, investigated the effects of APs in CHR-P.

McGorry and colleagues evaluated the efficacy of risperidone (1–2 mg/day) combined with a needs-based intervention and cognitive behavioral therapy (CBT), compared to a control group receiving the needs-based intervention alone, over a six-month period [31]. While both groups showed symptomatic improvement, the reduction in transition risk observed in the risperidone group was limited to the treatment phase and diminished after discontinuation. Additionally, 12% of patients treated with risperidone reported extrapyramidal side effects, specifically stiffness. In a subsequent 12-month study [32], no statistically significant differences in transition rates were observed between the three groups (CBT + risperidone, CBT + placebo, supportive therapy + placebo). The authors concluded that supportive therapy may be an effective initial intervention with fewer side effects.

A double-blind, randomized controlled trial [33] studied the effects of olanzapine (mean dose 10.2 mg/day) versus placebo in patients who met the criteria for prodromal syndrome. The findings suggest that olanzapine significantly improved positive symptom levels. However, its use was also associated with substantial weight gain and an increased risk of treatment discontinuation.

Similarly, McGlashan and co-authors [34], in a double-blind randomized trial, compared the effects of olanzapine (5–15 mg/day). Although the conversion rate to psychosis was higher in the placebo group, the difference did not reach statistical significance. Nevertheless, participants treated with olanzapine demonstrated a significant improvement in positive prodromal symptoms compared to those receiving a placebo.

In an open-label trial conducted by the German Research Network on Schizophrenia, Ruhrmann and colleagues randomly assigned individuals with CHR-P to receive a needs-focused intervention either with or without amisulpride (mean daily dose: 118.7 mg) [35]. The amisulpride group showed significant improvement in both attenuated and full-blown psychotic symptoms, as well as enhanced global functioning at 12 weeks. However, 81% of patients treated with amisulpride developed elevated blood prolactin levels [36].

The effect of aripiprazole was tested in an open-label study by Woods and colleagues [37]. The authors demonstrated that aripiprazole improves attenuated positive symptoms scores at 8 weeks, but akathisia was frequently experienced.

A 12-week open-label prospective study [38] examines the speed of response, doses, and safety of treatment with SGAs (mainly olanzapine, aripiprazole, and risperidone). The study compared individuals with CHR-P to those with first-episode schizophrenia (FES) and multi-episode schizophrenia (MES). The CHR-P group exhibited a significantly greater improvement in PANSS scores compared to the FES and MES groups; however, specific data on positive symptomatology were not reported. Additionally, the CHR-P group received significantly lower modal doses of SGAs (chlorpromazine equivalent: 183 mg/day, SD = 201.1) compared to the other two groups.

Liu and colleagues [39] reported a favorable clinical response to low-to-moderate doses of aripiprazole in individuals at CHR-P. All participants demonstrated significant improvement in PANSS positive symptom scores, aligning with previous findings on the psychopathological progression of the prepsychotic state from the SOPRES study in Taiwan [40,41] as well as results reported by Kobayashi et al. [42].

In a naturalistic observational longitudinal study from the SHARP program, the authors examined whether initiating AP treatment during the CHR-P phase yields better outcomes than initiating at the onset of FEP [43]. While both the CHR-P and FEP groups treated with comparable AP regimens demonstrated reductions in positive symptoms, individuals who received APs during the FEP phase showed significantly greater symptomatic and functional improvements, as well as higher remission rates. Consistent with other findings [43,44], the authors concluded that APs may not be an appropriate first-line intervention for CHR-P individuals. Early pharmacological intervention during this premorbid phase may contribute to poorer long-term outcomes, potentially due to adverse effects, the impact of stigma, and interference with key neurodevelopmental and psychosocial processes critical to recovery in adolescents and young adults.

Finally, a retrospective analysis was also conducted on 127 participants from the SHARP cohort by Zeng and colleagues [45]. The study focused on neurocognitive functioning in CHR-P individuals, comparing aripiprazole, olanzapine, and no antipsychotic treatment, with findings detailed in the cognitive section of this paper. Notably, the aripiprazole group showed significant reductions in positive, disorganization, and general symptoms at both 8 weeks and 1 year, while the olanzapine group showed improvements in positive and general symptoms at 1 year.

In conclusion, AP use in CHR-P for positive symptom treatment should be carefully considered, weighing short-term benefits against potential risks. Further research is needed to guide individualized, stage-specific interventions and to assess long-term efficacy.

## 3. Antipsychotics’ Effects on Negative Symptoms in CHR-P Individuals

Negative symptoms represent a core feature of SCZ [46,47]. According to the NIMH-MATRICS consensus statement [48], they consist of blunted affect, alogia, asociality, anhedonia, and avolition, and they have been proposed as a distinctive SCZ domain with proper pathophysiological and therapeutic implications.

Youth at CHR-P frequently present with a wide range of negative symptoms that have been reported to predict full conversion to psychosis [49] and to significantly reduce patients’ quality of life [50,51,52,53]. So, the need for treatments targeting negative symptoms in CHR-P is clear and unquestioned.

Current treatment guidelines for CHR-P recommend psychotherapies, such as cognitive behavioral therapy (CBT) and focused family therapy, as first-line interventions [16]. APs primarily target positive symptoms, but appear to have limited efficacy on negative symptoms in both adults with psychosis and CHR-P youth when compared to placebo [54].

A network meta-analysis was conducted to evaluate the effects of various interventions on negative symptoms in CHR-P populations; however, no treatment demonstrated clear efficacy or effectiveness [55]. A major limitation of the analysis was that most included studies were not specifically designed to target negative symptoms, and the heterogeneity of assessment tools further complicated the interpretation of results. Furthermore, results cannot disambiguate between primary negative symptoms (pathophysiological mechanisms of SCZ) and secondary negative symptoms (due to other mechanisms such as depression or antipsychotic side effects). In this context, it is important to note that CHR-P youth frequently present with high rates of comorbid depression and anxiety [56], which may overlap with or be mistaken for negative symptoms, such as anhedonia [38]. Although no intervention in Devoe and colleagues’ meta-analysis [55] showed robust superiority, some APs (e.g., amisulpride and olanzapine) showed potential signals of benefit over psychosocial interventions. However, these findings did not reach statistical significance. As previously noted, two studies [31,32] investigated the effect of low-dose risperidone (1–2 mg/day) combined with a needs-based intervention and CBT in a total of 146 participants. Risperidone was not associated with a significant reduction in negative symptoms at either 6 or 12 months of follow-up. Similarly, in a double-blind randomized controlled trial, olanzapine did not demonstrate greater efficacy than placebo in reducing negative symptoms [34].

Woods and colleagues [37] examined the short-term efficacy and safety of aripiprazole treatment in people with a psychosis prodrome, finding a reduction in negative symptoms on the SOPS subscale.

In the study by Ruhrmann and colleagues [35], amisulpride, combined with a needs-focused intervention, appears to be more effective in reducing negative symptoms than only need-based interventions.

Overall, three studies investigating aripiprazole [37,39,42], comprising a total of 61 participants, reported pre-/post-treatment assessments of negative symptoms. In two of these studies, aripiprazole was associated with a significant reduction in negative symptoms; however, the absence of a control group limits the interpretability of these findings. Walker and colleagues [57] investigated the relationship between antipsychotic and antidepressant use and the severity of prodromal symptoms at baseline and after six months of follow-up in a cohort of 191 individuals at CHR-P. Among the 48 patients treated with SGAs, no significant association was found between AP use and changes in negative symptom severity over time. Washida and colleagues [38] investigated response speed, dosage, and safety of SGA treatment in CHR-P patients compared to patients with SCZ. Their findings indicated that a lower modal dose of SGAs was sufficient for clinical improvement in the CHR-P group. However, the study did not employ any specific scale for the assessment of negative symptoms.

Tsujino and colleagues [41] reported similarly non-significant results regarding the efficacy of perospirone, a combined serotonin (5-hydroxytryptamine 5-HT)/dopamine antagonist and a 5-HT1A receptor agonist, in treating negative symptoms in a sample of 11 CHR-P individuals.

Only one study [58] investigated the use of ziprasidone in delaying or preventing conversion to psychosis, but no significant differences in negative symptoms were shown at follow-up.

Zhang and colleagues [44] analyzed a cohort of 517 CHR-P subjects and found no significant differences in terms of negative symptoms between the group of patients treated with APs and those not treated.

On the other hand, aripiprazole and olanzapine were both associated with a significant reduction in negative symptoms in Zeng et al.’s [45] study.

Finally, a recent study also investigated the effects of AP treatment on the disorganization domain in CHR-P individuals [59]. The inclusion of this research in the negative symptoms section, primarily for paper structural reasons, is supported by findings suggesting that disorganization in CHR-P is more closely linked to negative rather than positive symptoms. Biancalani and colleagues examined the longitudinal course of disorganization over a 2-year follow-up in CHR-P individuals. Disorganization was assessed using the eight-item factor model derived from Shafer and Dazzi’s PANSS meta-analysis (items P2, N5, N7, G5, G10, G11, G13, G15) [60]. The study found a significant reduction in disorganization severity over time in the AP-treated group, whereas a similar trend in the untreated group did not reach statistical significance. In conclusion, treatment options for negative symptoms remain inadequate. However, the study of negative symptoms in the prodromal state may provide insight into the underlying pathophysiological mechanisms in SCZ and lead to effective interventions [61,62].

## 4. Antipsychotics’ Effects on Cognitive Impairment in CHR-P Individuals

Cognition is a broad construct that can be defined as one’s ability to perceive, process, attend to, and remember information [63]. From Kraepelin’s early definition of SCZ as dementia praecox, impaired cognition has always been a core feature of this psychiatric condition [64]. Moreover, altered cognitive performances have been proven to predict poor functional outcomes [65,66] and a higher likelihood of relapse [67]. Abnormal neurodevelopment is, in fact, believed to be the cause of poor cognitive abilities among people with SCZ. These cognitive impairments appear to begin early during prenatal life, increase during childhood and adolescence, and consolidate during adulthood, according to the continuum of SCZ [68]. In other words, one could expect to observe milder imbalances in young patients with CHR-P and a major impact on cognition in later stages of chronic SCZ. To this end, a recent article showed how CHR-P subjects performed significantly better compared to the chronic SCZ group and significantly worse compared to healthy controls in multiple cognitive domains [69].

Since cognition deeply weighs on vocational and social functioning, ranging from academic and working performances [70] to mating abilities [71], clinical psychiatrists are required to establish appropriate treatments to prevent this impairment from further worsening [72,73]. Although APs are considered the standard of care for the treatment of SCZ, substantial evidence highlights their association with a wide range of side effects and medical complications [74]. To this end, although a large number of studies have analyzed any possible negative or positive effect, the relationship between APs and cognition among patients with SCZ still remains obscure and controversial [75].

From a historical perspective, first-generation APs (FGAs) have long been considered to exert a null or even negative effect on cognition, whereas their newer counterparts, SGAs, have also been introduced with the promise of improving cognitive functioning [76,77]. Since their introduction, SGAs have been associated with cognitive benefits across several domains. Clozapine, aripiprazole, and olanzapine have been shown to enhance verbal fluency, vigilance, selective attention, memory, and executive functioning [78,79,80]. Quetiapine has demonstrated promising effects on self-reported cognitive functioning [81], while risperidone has been linked to modest improvements in delayed recall [80]. Nonetheless, the superiority of SGAs in cognition was questioned by further studies, where the authors reported modest effect sizes for this class of APs and mostly comparable to FGAs [82].

These mixed findings may be explained by the fact that multiple confounders, such as dose of APs, improvement in symptoms, adherence to medication, and stage of intervention, can affect the relation between APs and cognition [75]. For instance, evidence from previous studies has shown an inverse relation between AP doses and cognitive abilities, with a higher posology, together with multiple-drug regimens, being associated with worse cognitive outcomes [83]. The same study also reported that AP dose and duration of treatment may have a combined effect, with findings showing that time-dilated regimens together with a high AP posology tend to negatively impact multiple cognitive domains, such as recall performance and verbal learning. Consistently, evidence on this topic has also shown improvements in language, attention, and memory when reducing the AP dosage [84].

Mixed findings have also been found when considering psychotic symptoms. On the one hand, an early study highlighted a direct association between cognitive abilities, such as speed of processing, and improvements in negative symptoms during AP therapies [85]. On the other hand, other authors reported that improvements in executive functioning are not related to improvements in positive symptomatology when patients were treated with drug regimens [86].

From a medication adherence perspective, patients who did not adhere to their AP drug schedule have been reported to have worse cognitive performance compared to those who were taking their medications adherently [87]. To this end, another study pointed out that verbal learning, memory, executive function, sustained attention, and visuomotor speed were significantly improved in patients who switched from oral medicines to long-acting injectable risperidone [88].

Among these different variables, the stage of psychosis also seems to play a key role in the association between APs and cognition. Several authors indeed corroborated the idea that the earlier the stage is, the greater improvements in cognition might be seen when establishing an AP treatment. In young patients with a FEP, improvements in cognitive abilities have been observed three months after the beginning of the treatment and continue in the following three [89] and five years of follow-up [90].

Although a substantial body of literature exists on FEP and chronic SCZ, evidence on the relationship between APs and cognition in CHR-P individuals remains limited, particularly regarding their effects on specific neurocognitive domains.

At the current state of the art, only seven studies have been conducted on this topic, showing mostly negative results.

In a controlled trial, Woods and colleagues [37] examined the neuropsychological effects of 8 weeks of aripiprazole treatment in CHR-P individuals. The results revealed a heterogeneous pattern: while improvements were noted in attention and working memory (e.g., CPT D-prime and 2-back tasks) and a trend toward enhanced executive functioning (fewer WCST perseverative errors), other cognitive measures showed no improvement or even decline, with a significant deterioration observed in semantic fluency. These findings suggest that aripiprazole did not consistently enhance cognition across domains in this population.

In an observational study, Bowie and colleagues analyzed a cohort of 70 subjects with CHR-P divided into four groups: no medication (*n* = 27), antidepressant (*n* = 15), APs (*n* = 11), and healthy controls (*n* = 17). The authors showed that the AP group had a worse performance in verbal learning and sustained attention, compared to the antidepressant group, but not compared to the other two [91].

Carrion et al. highlighted that treatment with APs did not influence any cognitive outcome measure in CHR-P subjects who did not develop psychosis further [92].

In a 2-year observational study, Pellizza and colleagues analyzed social cognition in CHR-P. The regression analysis showed a significant direct association between the dose of APs and the clinical severity in social cognition at 1-year follow-up (b = 0.330; *p* = 0.007) and a positive trend at 2-year follow-up (b = 0.357; *p* = 0.066) [93].

A retrospective analysis was conducted on 127 participants from the SHARP cohort, who were categorized into three groups based on treatment status (those receiving aripiprazole, those receiving olanzapine, and those not receiving any AP medication) [45]. The study has some limitations, notably the greater baseline symptom severity in the AP-treated groups. Nonetheless, the AP-naive group consistently outperformed treated groups, aligning with evidence suggesting that APs may not improve, and could worsen, cognition in CHR-P individuals. Notably, this study employed both ANCOVA and LMMs to evaluate the neurocognitive impact of AP treatments. ANCOVA, adjusting for baseline scores and treatment duration, showed domain-specific cognitive changes, with the AP-naive group demonstrating superior improvements in reasoning, problem-solving, and social cognition. Aripiprazole improved attention more than olanzapine, which showed the least cognitive benefit. LMMs confirmed these findings longitudinally, with the AP-naive group outperforming olanzapine across domains and aripiprazole in processing speed, verbal learning, and reasoning. Aripiprazole also outperformed olanzapine in visual learning, with significant interaction effects observed between groups.

Woodberry et al., in their sample of 53 CHR-P and 32 healthy controls, found that AP treatment was significantly correlated with lower baseline memory performance [94].

Finally, Zhang et al. [95] conducted a longitudinal study to examine the AP effects on cognitive function and clinical outcomes in adolescents and adults with CHR-P. Cognitive functions have been measured using the Measurement and Treatment Research to Improve Cognition in Schizophrenia (MATRICS) consensus cognitive battery (MCCB) [96]. Among 231 CHR-P treated with APs (olanzapine-equivalent dose mean dosage 8.5 (SD ± 6.1) mg/day; mean duration of administration 36.2 (SD ± 20.0) weeks), 161 completed a 1-year follow-up. Adolescents receiving APs (Adolescent-APs+) had more severe symptoms, worse functioning, and poorer cognitive performance at baseline compared to untreated peers (Adolescent-APs−). They also showed a significantly higher risk of transition to psychosis, less improvement in cognition, poorer performance on the BVMT-R and Category Fluency tests compared to Adolescent-APs−, and lower functioning at follow-up. In contrast, no significant differences in cognitive or clinical outcomes were observed among adults CHR-P, although a non-significant trend toward reduced conversion was noted in the Adult-APs+ group. Although data from these observational studies seem to point toward a non-positive effect of APs on cognition in CHR-P subjects, several limitations prevent consistent conclusions from being drawn. Firstly, data are collected in a naturalistic fashion, without any interventional design. Hence, no clear cause-and-effect mechanism could be addressed. Secondly, the study cohorts are small and not randomized, meaning that many variables could bias any hypothesis. Lastly, none of these works have analyzed single APs. This means that poor considerations can be made about the pharmacodynamic profile of these drugs. For instance, a recent work highlighted how APs with anticholinergic properties significantly affect cognitive function in patients with FES [97]. This would, in fact, guide clinicians to choose other compounds with less muscarinic antagonism. Lastly, a recent systematic review highlights that neurocognitive abilities may already be impaired before flourishing symptomatology begins and then remain stable during the transition to psychosis in adolescents with CHR-P. Consequently, this perspective minimizes the significance of AP medications as cognitive disruptors [98]. The principal characteristics of the studies reviewed are summarized in Table 1, while the main findings across symptom domains are presented in Table 2.

## 5. Discussion

Given that there is a lack of guidelines and consensus for AP treatments for positive, negative, and cognitive symptoms in patients with CHR-P, the scope of our narrative review was to summarize the current knowledge on the topic.

Possibly due to their greater clinical recognizability, the clearest treatment guidelines for AP use in CHR-P individuals pertain to attenuated positive symptoms. APs are intuitively expected to be most effective for these symptoms, given their presumed linear progression toward full-blown psychosis. Currently, the EPA recommends AP drugs in individuals as a second-line treatment only after CBT, and they are not intended to prevent conversion to frank psychosis but rather to achieve stabilization of positive symptoms to allow psychological treatment [16]. In fact, some later studies suggest that AP drugs are a risk factor for conversion to frank psychosis [44,99]. This may be due to selection bias since groups receiving AP drugs may differ compared to patients without treatment. Factors related to positive symptoms, such as abnormalities in thought and perception, with consequently a higher risk of conversion, could influence the physician’s choice of AP treatment [100,101]. To date, high-quality RCTs on the efficacy of APs in preventing frank psychosis or improving symptoms are still lacking [30]. For now, the EPA guidance should be applied and adapted to the individual.

Few studies have investigated the treatment of negative symptoms in CHR-P, possibly due to the difficulty in detecting and comparing attenuated negative symptoms and their tendency to vary over time. Based on available evidence, AP treatment is discouraged in attenuated negative symptoms. However, it may be helpful in specific settings as a second-line treatment option after careful individual consideration. Intuitively, this would make sense and would be consistent with our current knowledge of patients with SCZ experiencing negative symptoms [102]. Given the observed improvement trends associated with certain SGAs (e.g., olanzapine and amisulpride) [55], studies specifically examining and distinguishing the effects of AP treatment on primary versus secondary negative symptoms are warranted. Future research is encouraged to explore the diagnosis and treatment of attenuated negative symptoms in CHR-P individuals further. Fewer studies on cognitive symptoms in CHR-P have been published, and most studies are derived from the SCZ literature. This may be due, in part, to the challenges associated with detecting and evaluating subtle cognitive deficits in the CHR-P population, which are generally less pronounced than those observed in patients with full-blown SCZ. Interestingly, the available data on CHR-P, albeit based on low-quality observational data, contrasts with our understanding of treatment for patients with SCZ. In CHR-P adolescents, AP treatment appears to be associated with poorer cognitive recovery and clinical outcomes, highlighting the need for cautious evaluation before initiation. The discrepancy in terms of positive effects on cognitive domains between CHR-P and FES treated with APs may reflect selection biases [78,79,80,81] and underscores the need for further research to clarify these differential effects.

Finally, recent evidence suggests that AP prescriptions at baseline represent an early “warning flag”, showing increased risk of hospitalization during the first two years of treatment. However, over the longer term, baseline AP use appears to be associated with some positive outcomes, such as a reduced risk of service disengagement [103].

## 6. Conclusions

In conclusion, the use of APs as a first-line treatment in CHR-P is not recommended due to the limited evidence available and requires maximum attention to side effects and possible stigma they may cause.

We concur with the perspective expressed by De Lisi and colleagues [19], who emphasize the importance of a comprehensive assessment of CHR-P to more accurately evaluate the effectiveness of interventions, including AP treatment. Such assessment should encompass key clinical dimensions, including real-world functioning, persistent negative symptoms, diagnostic trajectories, psychosocial functioning, subjective psychological distress, quality of life, comorbidities, personal and social recovery, stigma, community integration, neurocognitive performance, and reduced mismatch negativity (MMN) amplitude. Moreover, in the original definition of UHR, the initiation of AP treatment was considered a functional equivalent of transition to psychosis [104]. To better understand the clinical trajectory of CHR-P individuals, studies should adopt a less permissive approach and ensure clearer differentiation between those with and without AP exposure at baseline [11,104]. This approach may aid in identifying specific factors and phenotypic profiles within CHR-P individuals that influence the course and prognosis of psychosis [105,106,107,108].

Finally, recent meta-analysis [109] has shown no robust or sustained effects of current active interventions on key outcomes in CHR-P, including transition to psychosis. These results underscore the need for novel approaches and therapeutic strategies in this population.

Additional studies with larger cohorts and RCTs on individual APs are necessary, as well as a division of the type of attenuated symptoms. Individual consideration should be taken in CHR-P subjects with rapid deterioration, such as suicidal behavior and aggression. In these patients, the use of SGA in low doses may be a valid option. However, there is no doubt that as a first-line treatment, CBT, rehabilitative approaches, and family interventions should be practiced [16,25,110].

## Figures and Tables

**Table 1:** Characteristics of CHR-P AP studies.

First Author, Year	Study Design	Sample Size	Age: Mean (SD)	CHR-P Criteria	Antipsychotics	Adjunctive Therapies	Positive Symptoms Tool	Negative Symptoms Tool	Cognitive Tool	Outcome Measured
McGorry et al., 2002 [31]	Single-blind RCT	59	20 (3.6)	CAARMS	Risperidone	CBT (all); antidepressants and benzodiazepines where appropriate	BPRSP	SANS	NR	Development of suprathreshold levels of psychosis
McGorry et al., 2013 [32]	Double-blind RCT	115	17.6 (2.6)	CAARMS	Risperidone	CBT; supportive therapy	BPRSP	SANS	NR	Conversion to psychosis
Woods et al., 2003 [33]	Double-blind RCT	60	18.2 (5.5)	SIPS/SOPS	Olanzapine	Individual and family psychosocial interventions; benzodiazepines for agitation and/or insomnia	SOPS Pos; PANSS Pos	SOPS Neg; PANSS Neg	NR	Reduction in prodromal symptomatology
McGlashan et al., 2006 [34]	Double-blind RCT	31	18.2 (5.5)	SIPS/SOPS	Olanzapine	NR	SOPS Pos; PANSS Pos	SOPS Neg; PANSS Neg	NR	Reduction in transition rates to full-blown psychosis
Ruhrmann et al., 2007 [35]	Open-label RCT	65	26.1 (6.1)	ERIraos	Amisulpride	Needs focused intervention; antidepressants and benzodiazepines where appropriate	PANSS Pos	PANSS Neg	NR	Reduction in prodromal symptomatology and improvement in functioning
Woods et al., 2007 [37]	Open-label, uncontrolled trial	15	17.1 (5.5)	SIPS/SOPS	Aripiprazole	Antidepressants and benzodiazepines where appropriate	SOPS Pos	SOPS Neg	CPT-IP; Letter-number sequencing; N-back; TMT (A and B); Stroop Color Word Test; AVLT; WCST; Semantic fluency; Phonemic fluency	Reduction in prodromal symptomatology
Washida et al., 2013 [38]	Open-label, uncontrolled trial	17	23.7 (4.4)	CAARMS	SGAs	Supportive psychotherapy, occupational therapy, antidepressants, and benzodiazepines where appropriate	PANSS Pos	PANSS Neg	NR	Improvement in psychopathology and functioning, and extrapyramidal side effects
Liu et al., 2013 [39]	Open-label, uncontrolled trial	11	21.5 (3.7)	CAARMS	Aripiprazole	NR	PANNS Pos	PANSS Neg	NR	Reduction in prodromal symptomatology
Tsujino et al., 2013 [41]	Open-label, uncontrolled trial	11	26.7 (6.5)	SIPS/SOPS	Perospirone	Antidepressants, mood stabilizers, or benzodiazepines where appropriate	SOPS Pos	SOPS Neg	NR	Reduction in positive prodromal symptomatology without causing severe adverse effects
Kobayashi et al., 2009 [42]	Open-label	36	23.4 (5.6)	SIPS/SOPS	Aripiprazole	NR	SOPS Pos	SOPS Neg	NR	Reduction in prodromal symptomatology and improvement in insight
Zhang et al., 2020 [44]	Naturalistic longitudinal study	309	19.8 (5.8)	SIPS/SOPS	SGAs	Antidepressants (fluoxetine-equivalent dose of 45.7 mg (SD = 38.4) in 131 individuals	SOPS Pos	SOPS Neg	NR	Conversion to psychosis
Zhang et al., 2021 [43]	Naturalistic longitudinal study	105	18.6 (5.1)	SIPS/SOPS	SGAs	Antidepressants (fluoxetine-equivalent dose of 22.4 mg (SD = 11.6) in 38 individuals	SOPS Pos	SOPS Neg	NR	Conversion to psychosis and poor functioning
Zeng et al., 2025 [45]	Observational study	127	19.4 (5.2)	SIPS/SOPS	Aripiprazole; Olanzapine	NR	SOPS Pos	SOPS Neg	MCCB	Improvement in psychopathology and functioning
Walker et al., 2009 [57]	Naturalistic study	191	18.65 (4.7)	SIPS/SOPS	Risperidone; Olanzapine; Quetiapine; Aripiprazole	Antidepressants	SOPS Pos	SOPS Neg	NR	Improvement in psychopatology
Woods et al., 2017 [58]	RCT	50	22.25 (NR)	SIPS/SOPS	Ziprasidone	Supportive Interpersonal Therapy	SOPS Pos	SOPS Neg	NR	Conversion to psychosis
Biancalani et al., 2025 [59]	Observational study	180	20 (NR)	SIPS/SOPS	SGAs	Antidepressants and benzodiazepines where appropriate	PANSS Pos	PANSS Neg	NR	Improvement in disorganization, psychopathology, and functioning
Bowie et al., 2012 [91]	Naturalistic study	70	16.44 (1.52)	SIPS/SOPS	SGAs	NR	SOPS Pos	SOPS Neg	CVLT total; CPT shapes; CPT digits; Verbal fluency; TMT (A and B); LNS	Neurocognitive functioning
Carrion et al., 2015 [92]	Nested case–control study	45	17.56 (1.35)	SIPS/SOPS	SGAs	Antidepressants	SOPS Pos	SOPS Neg	WISC-III/WAIS-R; CVLT; Digit Span; Letter-Number Span; WCST; COWAT; CPT-IP; TMT; WRAT-III	Cognitive impairments before and after psychosis onset
Pellizza et al., 2021 [93]	Observational study	97	18.84 (4.30)	CAARMS	Not specified—Not all UHR took AP medications	CBT	CAARMS Pos	CAARMS Neg	iGEOPT	Improvement in psychopathology and social cognition
Woodberry et al., 2013 [94]	RCT	85	16.1 (2.4)	SIPS/SOPS	Unspecified antipsychotic—Not all UHR took AP medications	Family-aided assertive community treatment	SOPS Pos	SOPS Neg	WRAT-III; WASI; CPT-IP-II; CVLT; Delis-Kaplan Executive; Verbal Fluency Condition; TMT; WCST-128; WMS-III (≥16) or WISC-IV (<16); Finger Tapping Test; B-SIT	Improvement in psychopathology and neurocognitive functioning
Zhang et al., 2024 [95]	Naturalistic longitudinal study	327	18.6 (5.1)	SIPS/SOPS	SGAs	Antidepressants where appropriate	SOPS Pos	SOPS Neg	MCCB	Improvement in neurocognitive functioning

Studies are listed in the table in the order in which they appear in the text. RCT, Randomized clinical trial; CAARMS, The Comprehensive Assessment of At-Risk Mental States; CBT, Cognitive behavioral therapy; BPRSP, Brief psychiatric rating scale psychotic subscale; SANS, Scale for the assessment of Negative Symptoms; NR, Not Reported; SIPS, Structured Interview for Psychosis-Risk Syndromes; SOPS, Scale of Prodromal Symptoms; SOPS Pos, Scale of Prodromal Symptoms Positive symptoms subscale; SOPS Neg, Scale of Prodromal Symptoms Negative symptoms subscale; PANSS, Positive and Negative Syndrome Scale; ERIraos, Early Recognition Inventory; CPT-IP, Continuous Performance Test-Identical Pairs version; TMT, Trail Making Test; AVLT, Auditory Verbal Learning Test; WCST, Wisconsin Card Sorting Test; SGAs, Second Generation Antipsychotic; MCCB, MATRICS Consensus Cognitive Battery; CVLT, California Verbal Learning Test; LNS, Letter-Number Sequencing; WISC-III, Wechsler Intelligence Scale for Children; WAIS-R, Wechsler Adult Intelligence Scale-Revised; COWAT, Controlled Oral Word Association Test; WRAT-III, Wide Range Achievement Test; iGEOPT, Italian GEOPTE Scale; WASI, Wechsler Abbreviated Scale of Intelligence; B-SIT, Brief Smell Identification Test.

**Table 2:** Key points related to each symptom domain.

Symptom Domains	Key Point Messages
Positive	CHR-P individuals may present with positive symptoms requiring intervention, but APs are not recommended as first-line treatment. They are reserved for severe or deteriorating cases. Clinical trials on SGAs showed short-term improvements in positive symptoms, though many were associated with adverse effects (e.g., weight gain, extrapyramidal symptoms, prolactin increase). Results from the SHARP program and other studies suggest that APs may be less effective when initiated during the CHR-P phase compared to the first-episode psychosis phase, with poorer functional and symptomatic long-term outcomes. Although both pharmacological and psychological treatments appear to reduce positive symptoms in the short term, the long-term benefits remain uncertain, highlighting the need for cautious and personalized treatment strategies.
Negative	Negative symptoms are prevalent in CHR-P individuals, predict conversion to psychosis, and significantly impact quality of life, yet remain insufficiently targeted by current treatments. Few studies have specifically assessed the efficacy of APs on negative symptoms in CHR-P, and existing findings are limited by methodological heterogeneity and lack of dedicated measures. Results are mixed: some studies report modest improvements (especially with aripiprazole, olanzapine, and amisulpride), but overall evidence remains inconclusive, with no consistent superiority over psychosocial interventions. Future research should include large-scale clinical trials explicitly designed to evaluate the impact of APs on negative symptoms in CHR-P populations.
Cognitive	No clear consensus has been reached on the relation between antipsychotics and cognition among people with SCZ. Although scarce findings on CHR-P are available, antipsychotics seem to exert a detrimental effect in multiple cognitive domains among these patients. Additional studies with larger and randomized clinical populations and considering single antipsychotic profiles are needed to draw more consistent conclusions.

CHR-P—Clinical high risk for psychosis. SCZ—Schizophrenia.

## Abbreviations

5-HT	5-hydroxytryptamine
APs	Antipsychotics
BS	Basic Symptoms
CBT	Cognitive Behavioral Therapy
CHR-P	Clinical high risk for psychosis
DSM-5-TR	Diagnostic and Statistical Manual, Text Revision
FES	First Episode Schizophrenia
FGAs	First-Generation Antipsychotics
MES	Multi-Episode Schizophrenia
SGAs	Second-Generation Antipsychotics
SCZ	Schizophrenia
UHR	Ultra-high risk
